# Valuable Table

**Published:** 1880-03

**Authors:** 


### VALUABLE TABLE,

CONTAINING the number of pounds in a bushel of the different articles named:

| | | |
|---|---|---|
| Of Bran . . . | Twelve | Pounds. |
| Of Blue Grass . . . | Fourteen | do |
| Of Shorts . . . | Eighteen | do |
| Of Dried Apples . . | Twenty-five | do |
| Of Oats, . . . . | Thirty-two | do |
| Of Dried Peaches . . | Thirty-three | do |
| Of Hemp Seed, . . | Forty-four | do |
| Of Timothy Seed . . | Forty-five | do |
| Of Castor Beans . . | Forty-six | do |
| Of Barley, . . . | Forty-eight | do |
| Of Flax Seed . . | Fifty-six | do |
| Of Rye . . . . | Fifty-six | do |
| Of Shelled Corn . . | Fifty-six | do |
| Of Onions . . . | Fifty-seven | do |
| Of Wheat . . . | Sixty | do |
| Of Clover Seed, . . | Sixty | do |
| Of Mineral Coal . . | Seventy | do |
| Of Salt . . . . | Seventy-five | do |
| Of Corn on the Cob, . | Seventy-five | do |

